# Physiological Changes and Differential Gene Expression of Tea Plants (*Camellia sinensis* (L.) Kuntze var. *niaowangensis* Q.H. Chen) Under Cold Stress

**DOI:** 10.1089/dna.2021.0147

**Published:** 2021-07-15

**Authors:** Ying Wang, Yan Li, Jihong Wang, Zhun Xiang, Peiyu Xi, Degang Zhao

**Affiliations:** ^1^ The Key Laboratory of Plant Resources Conservation and Germplasm Innovation in Mountainous Region (Ministry of Education), Institute of Agro-Bioengineering and College of Life Sciences, Guizhou University, Guiyang, China.; ^2^ Guizhou Province Institute of Biology, Guizhou Academy of Sciences, Guiyang, China.; ^3^ The Application Center for Plant Conservation Technology, Guizhou Academy of Agricultural Sciences, Guiyang, China.

**Keywords:** *Camellia sinensis* (L.) Kuntze var. *niaowangensis* Q.H. Chen, cold stress, physiological response, gene expression

## Abstract

Low temperature is an important factor that affects the growth and reproduction of tea plants [*Camellia sinensis* (L.) Kuntze]. In this study, Yunwu Tribute Tea cutting seedlings [*Camellia sinensis* (L.) Kuntze var. *niaowangensis* Q.H. Chen] were subjected to different low-temperature treatments in Guizhou Province, China, and the changes in physiological indicators of the leaves were measured to investigate the physiological response and cold tolerance of this variety. Under cold stress, the peak of antioxidant enzyme activity appeared on the third day of treatment at 1°C, indicating that Yunwu Tribute Tea could improve the resistance to cold stress through an increase in enzyme activity within a low-temperature range. However, after 3 days treatment at 1°C, the tolerance of plant had been exceeded; the ability to resist cold stress disappeared, and enzyme activity decreased. When the temperature or duration of stress exceeded the maximum tolerance of the plant, the synthesis of soluble substances decreased in concert with their protective effects. Under cold conditions, Yunwu Tribute Tea could increase the production of abscisic acid growth inhibitors and reduce those of indoleacetic acid, gibberellin, and other growth promoting substances to manage cold stress by regulating the balance of growth regulators in the plant. Five differential genes were screened as candidate genes from the Yunwu Tribute Tea cold stress transcriptome (DW, 1°C) for fluorescence quantitative analysis. The results showed that the changes in levels of expression of these genes under continuous cold stress significantly positively correlated with the corresponding physiological indicators. Nevertheless, the levels of expression of the Yunwu Tribute Tea polyphenol oxidase (*PPO*) gene and the gibberellin 3β-dioxygenase gene (*G3O2*) were reversely inhibited under cold stress. The result was consistent with the corresponding physiological indicators, and it provides a basis for the study of cold resistance mechanisms in tea plants.

## Introduction

Tea tree (*Camellia sinensis*) is an important cash crop, which is native to the tropics and subtropics, that prefers warm and wet conditions. Low temperature is an important factor that affects the growth and reproduction of tea plants. Extreme low temperature in the winter and frost in the early or late spring often cause damage to tea plants and restrict the development of tea industry (Zou *et al.*, 2008; Zhu *et al.*, 2013). The damage might result in curliness, brown, burn on young shoot and a reduction in production, or even fall of branch leaves, decline of growing, or death of plant at worst. The low-temperature freezing injury has become one of the main limiting climatic factors for tea production, causing huge economic losses to tea growers every year. For example, in 2008, a large range of continuous low temperature, rain, snow, and freezing weather occurred in the middle and lower reaches of the Yangtze River and its south regions as well as the eastern part of southwest China, resulting in a production reduction of more than 15% in spring tea production, which is estimated to cause an economic loss of 10.5 billion yuan to the tea industry (Wu, 2008).

Owing to the instability of global temperatures in recent years, it is critical to prevent low temperatures that can damage to the growth of tea trees. Efforts to improve the cold resistance of tea have been increasing in intensity. The cold resistance of tea plant has potential genetic characteristics, and cold acclimation can induce the expression of this potential and exert maximal resistance to cold resistance. During the process of cold acclimation, changes occur not only to the physiological and biochemical processes of tea plants but also to many specific proteins and mRNAs that are induced by low temperatures.

A series of physiological and biochemical changes that occur in cold acclimation are the result of molecular regulation, which relies on a large number of signal transduction and regulatory pathways to readjust the expression of genes to change the metabolism and growth of the plant to adapt to cold stress (Chinnusamy *et al.*, 2004). Currently, studies on plant cold-resistance physiology primarily focus on membrane permeability, protective enzyme systems, and changes in the content of permeable substances (He *et al.*, 2008; Shen *et al.*, 2009; Yu *et al.*, 2010). The main function of the protective enzyme system is to prevent membrane peroxidation and reduce or prevent the damage of biological membrane under stress by removing H_2_O_2_ (Zhang *et al.*, 2013). Excellent osmotic adjustment substances not only have the biological effect of enhancing the resistance of tea plants to freezing damage but can also sensitively respond to low-temperature changes from late autumn to severe winter to early and late spring, which has a biostatistical correlation. When plants are subjected to stresses such as low temperature, the endogenous hormones of plants (e.g., abscisic acid [ABA] and gibberellic acid [GA]) are considered to be the initiators of plant expression of cold resistance genes (Weyers and Paterson, 2002) and play an important role in enhancing the resistance of plant to cold temperatures.

The fine varieties of Yunwu Tribute Tea (*C. sinensis* (L.) Kuntze var. *niaowangensis* Q.H. Chen) are distributed in the Yunwu Mountain, Guizhou Province, China. Currently, most research on Yunwu Tribute Tea has focused on its biological characteristics (Liu *et al.*, 2012), propagation technology (Wang *et al.*, 2013), and the quality of tea produced from it (Wang *et al.*, 2011). The interaction of osmolytes, antioxidases, and phytohormones in tea plants under low temperatures is not well understood. There are few reports on the physiological response mechanisms of this cultivar to low temperatures. The study of cold resistance of Yunwu Tribute Tea for the utilization of its germplasm resources, introduction of cold resistance breeding, and improvement of technology to improve its resistance to cold temperatures are of substantial theoretical and practical significance.

Although some physiological, biochemical, and genetic markers of cold tolerance have been proposed in some tea plant genotypes (Hao *et al.*, 2018), many mechanisms remain unclear, since resistance to cold is related to structural, biochemical, and genetic regulation. Studies have shown that plants can actively accumulate a number of amino acids, sugars, and inorganic ions that play an important role in the stress response (Hildebrandt *et al.*, 2015). The identification of such metabolites and their functions is important to fully understand the mechanism of resistance to frost in tea plants (Li *et al.*, 2019).

In this study, 1-year-old Yunwu Tribute Tea plant cutting seedlings were subjected to cold stress and measured the various physiological indicators of leaves. This enabled the study of mechanism of Yunwu Tribute Tea to resist low-temperature stress from a physiological perspective. On this basis, the patterns of expression of five cold resistance-related genes of this variety of tea were analyzed under cold stress, and the correlation between cold resistance genes and the response of physiological indicators was analyzed. The results of physiological, biochemical, and gene expression studies were combined to analyze the molecular mechanism of cold resistance of Yunwu Tribute Tea.

## Materials and Methods

### Plant material, growth, and stress conditions

The test material was 1-year-old cutting seedlings of Yunwu Tribute Tea, taken from the nursery of the Tea Industry Office of the Agricultural Bureau of Yunwu Town, Guiding County, Guizhou Province, China. The experiment was conducted at the China Guizhou Province Institute of Biology in Guiyang, China, from July to August 2016. The plants were subjected to normal management techniques in a solar greenhouse under natural light. When the plants had grown 70–80 cm high, those in good health that had grown consistently were selected for the experimental treatment.

The seedlings were divided into four groups, each with six pots, and transferred to a light incubator on the first day of experiment. The temperature of light incubator was set to 10°C, 4°C and 1°C. The control temperature was 25°C, the light intensity was 10,000 lux, relative humidity 50%, and the photoperiod 16 h/8 h day/night. Functional leaves were selected for the measurement of physiological indicators at 1, 3, 5, 7, and 9 days of the stress treatment.

### Physiological measurements

The permeability of cell plasma membrane was determined using the conductivity method (Kong and Yi, 2008). Superoxide dismutase (SOD) activity was measured by the inhibition of photoreduction of nitroblue tetrazolium (Li, 2000). Peroxidase (POD) activity was measured using the guaiacol method (Li, 2000). Catalase (CAT) activity was measured using the ultraviolet absorption method (Kong and Yi, 2008). Polyphenol oxidase (PPO) activity was determined using the catechol method (Li, 2000). The content of malondialdehyde (MDA) was determined by the thiobarbituric acid colorimetric method (Kong and Yi, 2008), soluble protein (SP) by the Coomassie Brilliant Blue G-250 method, free proline by the acid ninhydrin method, and soluble sugar by anthrone colorimetry (Li, 2000). The contents of endogenous hormones ABA, indoleacetic acid (IAA), and gibberellin were determined by the use of enzyme-linked immunosorbent assay (ELISA) kits that were purchased from the State Key Laboratory of Plant Physiology and Biochemistry, China Agricultural University, Beijing, China.

### RNA extraction and quantitative real-time polymerase chain reaction

The total RNA was extracted from cell samples by the addition of 1000 μL of TRIzol (Invitrogen, Carlsbad, CA) to the cell samples. The samples were fully mixed and incubated at room temperature for 30 min. A volume of 200 μL of chloroform was added, fully vortexed, and incubated at room temperature for 10 min. The samples were centrifuged at 12,000 *g* for 15 min at 2–8°C. After centrifugation, 400 μL of the supernatant was transferred to a fresh clean 1.5 mL tube, and 500 μL of isopropanol was added. The tube was inverted several times and incubated at 4°C overnight. The samples were centrifuged at 12,000 *g* for 10 min at 2–8°C, and the supernatant was discarded. A volume of 1000 μL of 75% ethanol was added and centrifuged at 7500 *g* for 5 min at 4°C. The ethanol was discarded, and 30 μL of ddH_2_O was added to dissolve the RNA after all of the ethanol had volatilized. The RNA was incubated on ice for 15–20 min and then quantified using a NanoDrop 100 (Thermo Scientific, Waltham, MA). The first strand of cDNA was synthesized according to the manufacturer's instructions in the TRUEscript 1st Strand cDNA Synthesis Kit.

The reactions for the quantitative real-time polymerase chain reaction were as follows: 95°C 3 min; 95°C 10 s; 58°C 30 s +plate read; and 39 cycles of 95°C 10 s. After all of the components were added, the tubes were centrifuged at 6000 rpm for 1 min to keep all of the components in the bottom.

The primers (Table 1) were designed by Beacon Designer 7.9 (Premier Biosoft International, Diamond Heights Blvd, San Francisco) and synthesized by Chengdu Danfeng Technology Co., Ltd. (Chengdu, China). The amplification curve and melting peaks of each gene primers for qPCR analysis are shown in Supplementary Figures S1–S6.

**Table 1.** Forward and Reverse Primers Used for Quantitative Real-Time Polymerase Chain Reactions

Primer	Accession number	5*′ *to 3′	Length (bp)
*GPX*-F	JQ247186.1	F:CTGAGTGGAGAAGTAGTG	3314
*GPX*-R		R:GTGATGGAATTAAGTGGAAT	
*PPO*-F	FJ597757.1	F:CACAACCAACCTTCCCAACAAA	2122
*PPO*-R		R:TGCTTCTTGATTTCTTCGGTCTCT	
*P5CS*-F	KR363007.1	F:ATTGTTGATGATGTGTATGC	3454
*P5CS*-R		R:GGTCTTCTGTGATAATGCTA	
*NCED*-F	XM_012981017.1	F:GTAAGTCTCTGCTGTAAC	2510
*NCED*-R		R:CTGTCTCAATTCACTCTC	
*G3O2*-F	KF703744.1	F:CCTATGTTGACCACGAGAG	1340
*G3O2*-R		R:CGACCCTACTCACCATTC	

*G3O2*, gibberellin 3β-dioxygenase; *GPX*, glutathione peroxidase; *NCED*, 9-cis-epoxycarotenoid dioxygenase; *P5CS*, Δ1-pyrroline-5-carboxylate synthetase; *PPO*, polyphenol oxidase.

To understand the role of changes in the expression of candidate genes in the cold resistance of tea plant and the correlation with the corresponding physiological indicators described above, based on the previous cold stress transcriptome data, the levels of expression of five candidate differential genes (*GPX*, *PPO*, *P5C5*, *G3O2*, and *NCED*) of Yunwu Tribute Tea under cold stress were analyzed by real-time fluorescent quantitative PCR. The Pearson correlation between the physiological indicators and expression of corresponding genes was then analyzed. The calculation of the relative expression of target genes in each sample was performed automatically using the qPCRsoft 3.2s and Pfaffl methods simultaneously:


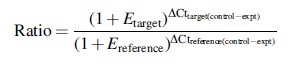


The relative gene expression of each sample was calculated using 2^−ΔΔCt^ with *ACTIN* as the internal reference.

### Statistical analysis

A one-way analysis of variance (ANOVA) followed by a *post hoc* Fisher's Least Significant Difference test was used to examine the significant differences between measurements at different stages. A Pearson correlation was used to analyze the correlation between physiological indicators, and the differences were considered to be significant at *p* < 0.05. Differences between samples in each treatment are indicated by letters (*p* < 0.05), while values with same letter did not differ from each other. The experimental data were analyzed for variance using SPSS 22.0 (IBM, Inc., Armonk, NY).

## Results

### Cytoplasmic membrane permeability

**FIG. 1.**
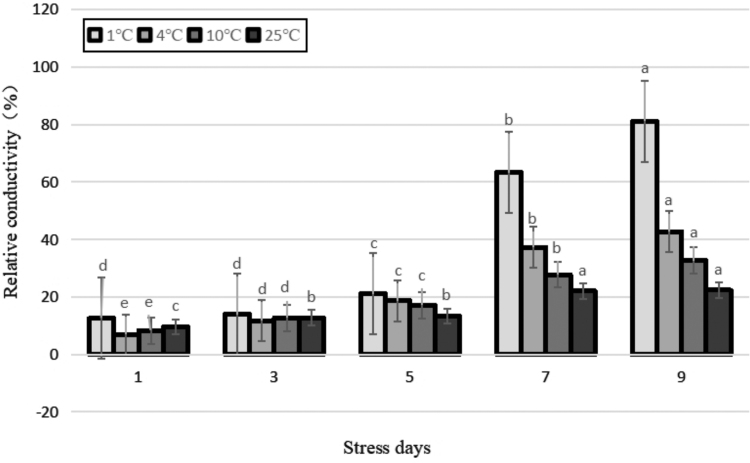
Effects of durative cold stress on relative conductivity. Data are displayed as mean values of the three replicates with standard error. The different letters indicate significant difference at 0.05 level among different samples.

With the decrease in temperature and the extension of treatment time, the relative conductivity (RC) of Yunwu Tribute Tea leaves tended to increase (Fig. 1), indicating that with the decrease in temperature and extension of treatment time, the damage to cell membrane of the tea leaves was intensified, and the membrane permeability gradually increased. However, at the initial stage of the stress treatment, the RC of each treatment increased relatively slowly. When the stress treatment was 5 days, the 10°C, 4°C, and 1°C treatments increased by 28.2%, 39.1%, and 57.4%, respectively, compared with the control. After 5 days of treatment, the RC of 10°C, 4°C, and 1°C treatments increased sharply, respectively, and at 7 days of stress treatment, it increased by 26.2%, 69.4%, and 187.5%, respectively, compared with the control. At the early stage of stress, the tea developed a self-protective function against adversity, and the changes in leaf RC were small. However, the later stages of stress exceeded the ability of the plant to resist them, resulting in a significant increase in the rate of electrolyte leakage, which could lead to the injury of tea by low temperature. During the whole stress process, the RC of the leaves treated at 1°C was significantly different from that at 10°C (*p* < 0.05).

### Lipid peroxidation

MDA is a major cytotoxic product of lipid peroxidation, and changes to its abundance represent the degree of lipid-membrane oxidation by oxidants such as free radicals (Ozkur *et al.*, 2009).

**FIG. 2.**
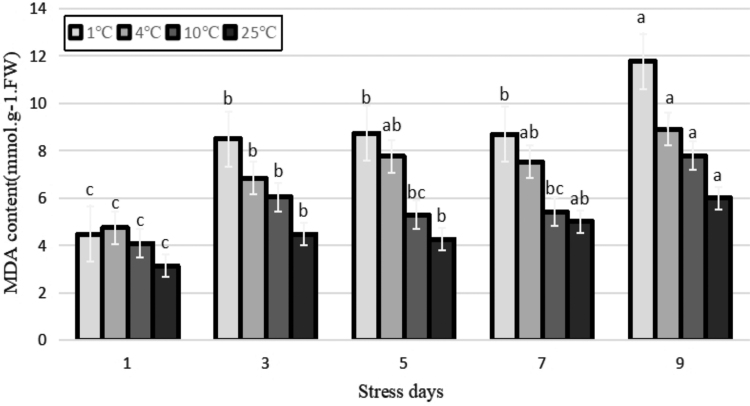
Effects of durative cold stress on the content of MDA. Data are displayed as mean values of the three replicates with standard error. The different letters indicate significant difference at 0.05 level among different samples. MDA, malondialdehyde.

Under cold stress, the MDA content of tea leaves increased (Fig. 2). The content of MDA increased in parallel with the temperature, which indicated that low temperatures caused an increase in the lipid peroxidation of cell membrane of the plant. The degree of increased membrane lipid peroxidation was directly proportional to the degree of cold stress. The MDA content of the control and 10°C treatments increased slightly with time, but the change was not obvious. The MDA content of both treatments at 4°C and 1°C continued to increase with the extension of duration of stress, indicating that the plants were unable to moderate the stress using their own physiological regulation. This low temperature damaged the leaf cell membrane, causing membrane lipid peroxidation. An ANOVA showed that the MDA content of leaves treated at 1°C was significantly different from those treated at 10°C (*p* < 0.01).

### Phytohormone response

**FIG. 3.**
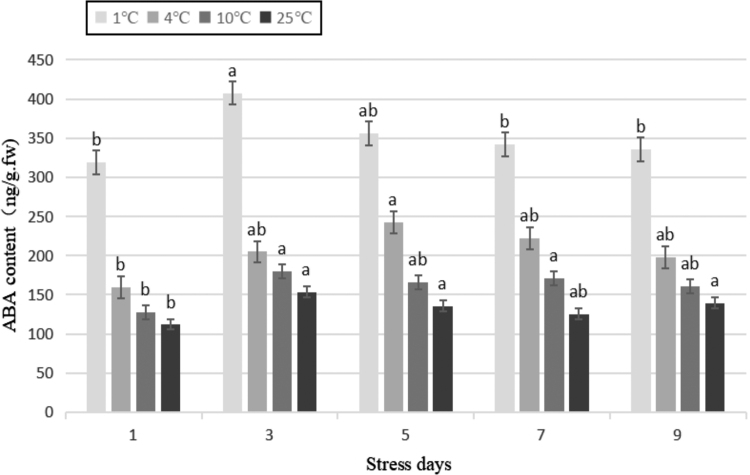
Effects of durative cold stress on the content of ABA. Data are displayed as mean values of the three replicates with standard error. The different letters indicate significant difference at 0.05 level among different samples. ABA, abscisic acid.

Under adverse conditions, the content of ABA increases significantly to adjust the adaptation of plant to stress (Gusta *et al.*, 2005). In this study, the ABA content of plant increased as the temperature decreased (Fig. 3), indicating that the endogenous ABA content increased after cold stress, serving as a protective response against low temperatures. With the prolongation of duration of stress, the ABA content of the plant increased and then decreased under different low-temperature treatments, with the highest content at 3, 5, and 3 days at 1°C, 4°C, and 10°C, respectively. It increased by 165.3%, 78.6%, and 16.8%, respectively, compared with the control. There were extremely significant differences among the temperature stress treatments (*p* < 0.01). This may be owing to the fact that the synthesis of ABA in plants is halted after its accumulation to a particular extent, and the cell membrane and protective enzyme system of plants have been damaged to some extent at the late stage of stress, resulting in a decrease.

**FIG. 4.**
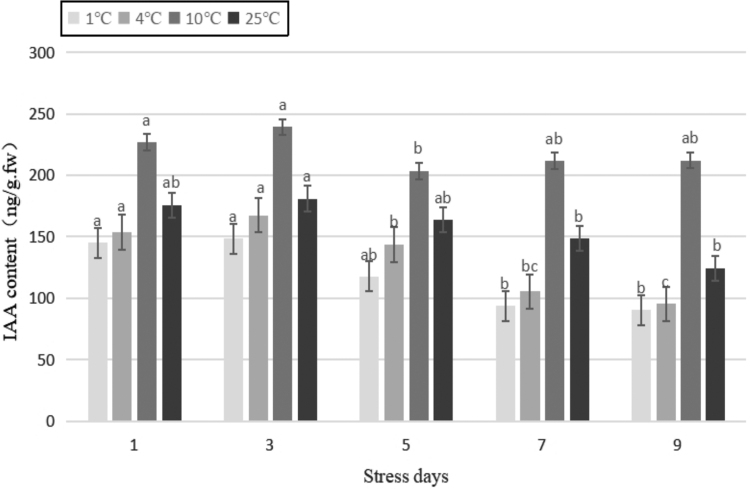
Effects of durative cold stress on the content of IAA. Data are displayed as mean values of the three replicates with standard error. The different letters indicate significant difference at 0.05 level among different samples. IAA, indoleacetic acid.

IAA is the most common type of auxin in plants and can promote cell division, elongation, and differentiation (Zhang *et al.*, 2013). As shown in Figure 4, compared with the control, the IAA content of the plant treated with the temperature of 1°C and 4°C decreased significantly (*p* < 0.01). Compared with the control, the IAA content at 10°C increased significantly (*p* < 0.01). Under the same stress temperature, with the extension of stress duration, the IAA content mostly increased at first and then decreased. After stress treatment at 4°C for 3 days, the IAA content decreased by 18% and 7.4%, respectively, compared with that of the control. The content of IAA increased by 32.12% compared with that of the control after stress treatment at 10°C for 3 days.

**FIG. 5.**
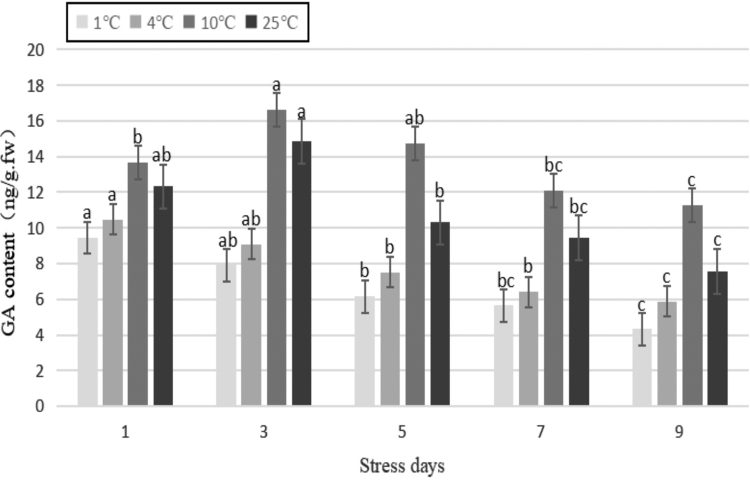
Effects of durative cold stress on the content of GA. Data are displayed as mean values of the three replicates with standard error. The different letters indicate significant difference at 0.05 level among different samples. GA, gibberellin.

GA is commonly found in plants and is one of the most important phytohormones for plant growth and development. As shown in Figure 5, with the extension of stress duration, the GA content of the plant decreased following treatment at 1°C and 4°C. The content of GA increased and then decreased following treatment at 10°C and 25°C. Compared with the control, the difference in GA content following treatment at 1°C and 4°C reached an extremely significant level (*p* < 0.01). After 9 days of stress, the content of GA decreased by 42.71% and 22.29%, respectively, compared with that of the control. The decrease in content of GA could inhibit plant growth and increase the resistance to cold, which indicated that the plants treated at 1 and 4°C could adapt to low temperatures by changing the content of GA in tissues.

IAA and GA are growth-promoting hormones and can therefore affect cell elongation, division, and differentiation and promote the growth and development of plants. The content of these hormones decreased under stress, which could reduce the growth of plant, and thus, reduce the damage caused by stress. As the temperature decreases, the tea plant may increase the synthesis of ABA and reduce the synthesis of IAA and GA to reduce the damage of low temperature, so that the plant enters or remains dormant.

### Changes in soluble sugars and proline content

**FIG. 6.**
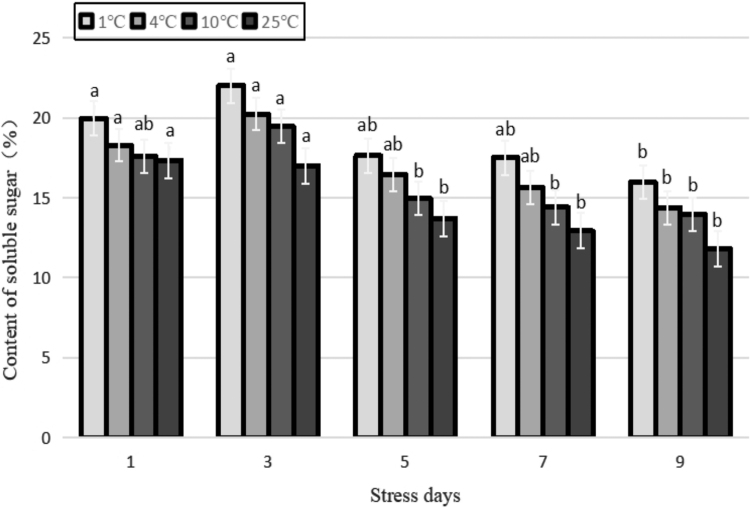
Effects of durative cold stress on the content of soluble sugar. Data are displayed as mean values of the three replicates with standard error. The different letters indicate significant difference at 0.05 level among different samples.

Compared with the control, the soluble sugar content of each low-temperature treatment increased significantly (Fig. 6). The ANOVA showed that the soluble sugar (SS) content under each low-temperature treatment differed substantially from that of the control (*p* < 0.01). As the stress duration was prolonged, the low-temperature treatments basically increased followed by a decrease, with the peak value appearing at 3 days at 10°C, 4°C, and 1°C after the stress treatment. Compared with the control, the soluble sugar content increased by 14.7%, 18.8% and 29.4%, respectively. It indicates that, under a short duration of cold stress, Yunwu Tribute Tea enhances its ability to resist cold by accumulating soluble sugars. However, as the duration of stress increases and the temperature decreases, the continuous low temperature causes damage to the plant, and the content of soluble sugar decreases.

**FIG. 7.**
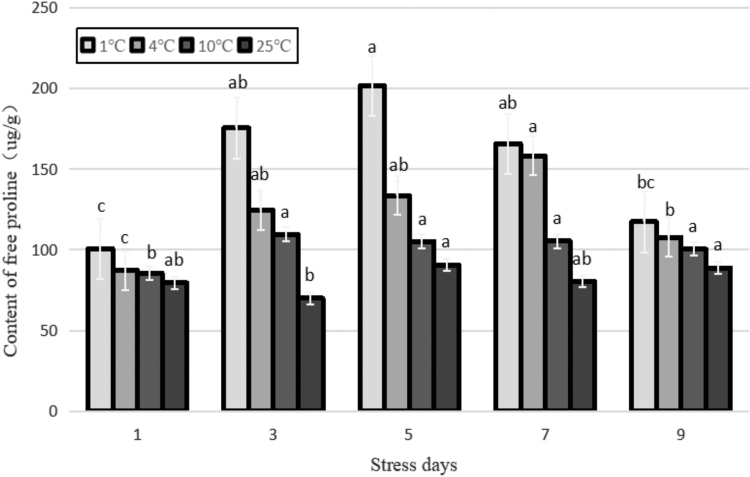
Effects of durative cold stress on the content of free proline. Data are displayed as mean values of the three replicates with standard error. The different letters indicate significant difference at 0.05 level among different samples.

The main role of free proline (Pro) in cold stress is considered to be as an osmotic adjuster and membrane stabilizer in the cell. Most studies have shown that its content strongly correlates with the resistance of plant to cold. Under tolerable levels of stress in plants, its content increases (He *et al.*, 2008). Figure 7 shows that compared with the control, the free proline content of each low-temperature treatment increased significantly, and the free proline content of the two low-temperature treatments at 4°C and 1°C differed significantly from those of the control (*p* < 0.01). As the stress duration was prolonged, the proline content of the treatment at 10°C barely changed. The free proline content of the two low-temperature treatments under 4°C and 1°C increased first and then decreased. The peak values appeared at 7 and 5 days after the stress treatment. Compared with the control, the content of free proline increased by 97.5% and 122.5%, respectively. This indicated that Yunwu Tribute Tea plants can temporarily balance the cold damage through their own physiological regulation under lower temperature treatment (1°C). However, as the duration of stress increased, the physiological regulation gradually decreased.

### Changes in SP

**FIG. 8.**
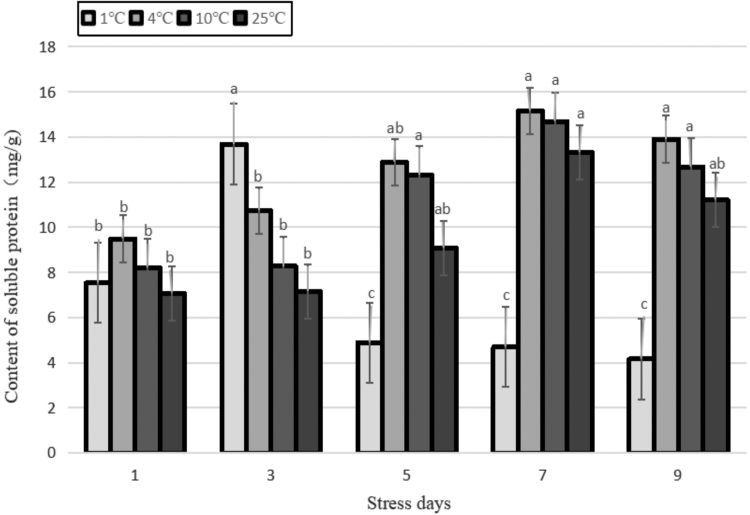
Effects of durative cold stress on the content of soluble protein. Data are displayed as mean values of the three replicates with standard error. The different letters indicate significant difference at 0.05 level among different samples.

SP is an important osmoregulatory substance and nutrient. Its increase and accumulation can improve the ability of cells to retain water and protect vital cellular substances and the biological membrane. Therefore, it is often used as one of the indicators for screening resistance. In this study, the content of SP increased with the decrease in temperature, indicating that the content of SP and temperature were negatively correlated within a particular range. The increase in protein content might be owing to the low temperature that promotes the synthesis of some new proteins. The analysis in Figure 8 shows that the SP content in the 1°C treatment was significantly different from that in the 4°C and 10°C treatments (*p* < 0.05). The SP content in the 1°C treatment first increased and then decreased, and it reached its highest value after 3 days of stress. It increased by 90.9% compared with that of the control, then dropped sharply before stabilizing, indicating that the plants cannot withstand continuous low temperatures. The rate of degradation of protein was faster than that of synthesis, and the content of SP of other treatments also showed a trend of increasing first and then decreasing, reaching the highest value after 7 days of stress. Compared with the control, the SP content increased by 10.3% and 13.7% at 10°C and 4°C, respectively.

### Changes to antioxidant activity

**FIG. 9.**
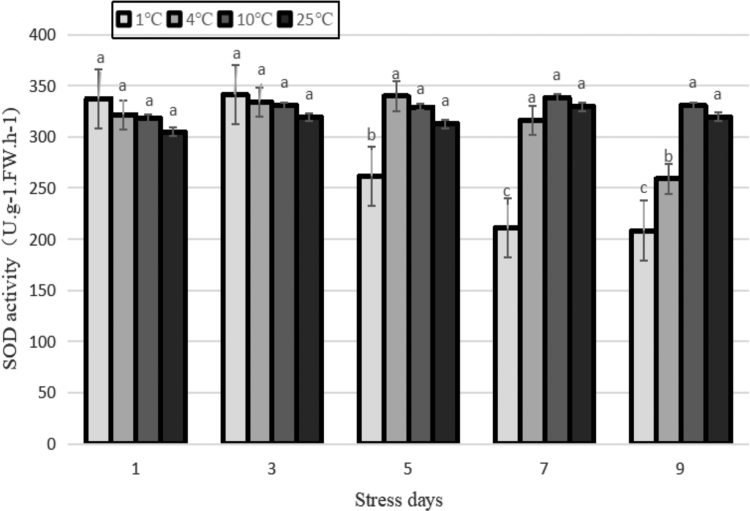
Effects of durative cold stress on SOD activity. Data are displayed as mean values of the three replicates with standard error. The different letters indicate significant difference at 0.05 level among different samples. SOD, superoxide dismutase.

Under different temperature gradient treatment conditions, the SOD activity of the tea leaves varied with time. As shown in Figure 9, there was no significant change in SOD activity with time for the control, and after the 10°C treatments, it showed a trend that increased slowly and then decreased sharply with time under treatments at 4°C and 1°C. The peaks of SOD activity appeared after 5 and 3 days, respectively. In the early stage of different stress treatments (before 3 days), the lower the temperature the larger the amount of SOD activity, indicating that the plants could enhance their resistance to cold by increasing the amount of SOD activity to remove excessive active oxygen free radicals in a short period of time. With the extension of cold stress time and the intensification of cold stress, SOD synthesis was also inhibited, and its activity started to decrease. During the entire stress process, SOD activity after treatment at 1°C was significantly different from those at 4°C and 10°C (*p* < 0.05).

**FIG. 10.**
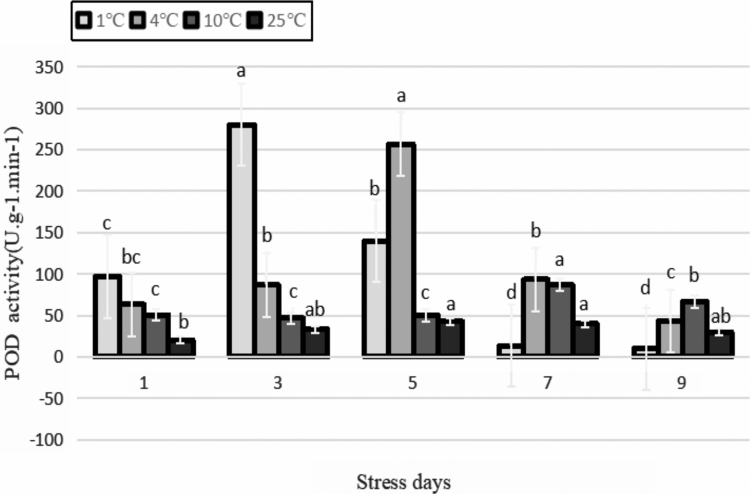
Effects of durative cold stress on POD activity. Data are displayed as mean values of the three replicates with standard error. The different letters indicate significant difference at 0.05 level among different samples. POD, peroxidase.

The role of POD in plants is complicated, and it is currently believed that the main role of POD in the protective enzyme system is to prevent membrane peroxidation by scavenging H_2_O_2_ and reducing or preventing damage to biofilms under adverse stress (Zhang *et al.*, 2013). As shown in Figure 10, in the initial stage of treatments under a different temperature gradient, the activity of POD increased as the temperature decreased, indicating that the plant slowed down the rate and degree of damage through a certain defensive response at the initial stage of cold stress. This reflects the ability of the plant to adjust and adapt to adversity. Overall, the POD activity of the leaves treated at 1°C and 4°C increased first and then decreased with the prolongation of stress duration. The highest peaks appeared at 3 and 5 days after stress, which increased by 7.4 times and 5.0 times, respectively, compared with that of the control and then decreased sharply. After that, the POD activity was lower than that of the control after 1°C stress treatment for 7 days. It can be seen that continuous low temperatures will cause an increase in active oxygen, such as superoxide free radicals, in Yunwu Tribute Tea, resulting in a decrease in POD activity. POD activity was not significantly different among the cold stress treatments during the entire stress process (*p* > 0.05).

**FIG. 11.**
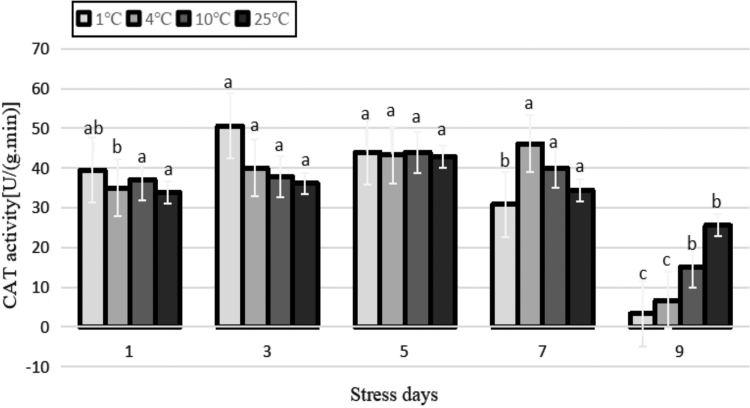
Effects of durative cold stress on CAT activity. Data are displayed as mean values of the three replicates with standard error. The different letters indicate significant difference at 0.05 level among different samples. CAT, catalase.

In the early stage of stress treatment (before 5 days), the CAT activity of each low-temperature treatment was greater than that of the control, with a pattern of increasing in parallel with the decrease of temperature, and the values were basically the same at 5 days. After 9 days of stress, the CAT activity of each low-temperature treatment was reduced to a level smaller than that of the control (Fig. 11). The CAT activity of leaves in each temperature treatment showed a trend of increasing and then decreasing with the extension of duration of stress. The highest peak in the 1°C treatment occurred on the third day of stress, and compared with the control, the CAT activity increased by 29.2%. The highest peak in the 4°C treatment occurred on the seventh day of the stress, which increased by 33.9% compared with the control. At the later stage of each cold stress treatment, the activity of CAT began to rapidly decline. This activity was significantly lower than the control at 9 days, indicating that with the increase in H_2_O_2_ content in the tissue of the leaves, the ability of CAT to scavenge decreased, resulting in an increased peroxidation in tissues and chilling damage. The activity of CAT was not significantly different among temperature treatments during the entire stress process (*p* > 0.05).

**FIG. 12.**
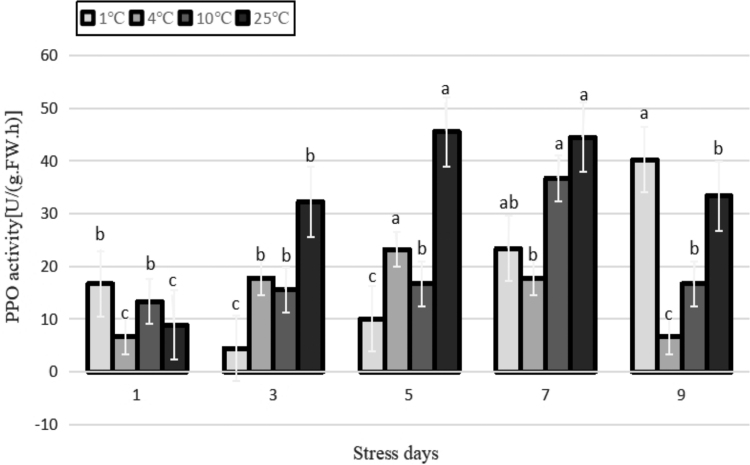
Effects of durative cold stress on PPO activity. Data are displayed as mean values of the three replicates with standard error. The different letters indicate significant difference at 0.05 level among different samples. PPO, polyphenol oxidase.

PPO is bound to the endosomal membrane and is inactive in its natural state. However, after homogenization or loss of tissues, PPO is activated, and thus, exhibits activity. PPO activity basically showed a trend of increasing and then decreasing in each temperature treatment throughout the stress process (Fig. 12). In the early stage of treatment at 1°C, the activity of PPO decreased owing to the reduction of photosynthesis, accumulation of organic matter, and phenolics in the plant. However, in the later stage of treatment, PPO was activated, and its content increased after the loss of tissues owing to the prolonged cold stress. In the initial stage of other temperature stress treatments, to resist the low temperature, plants themselves increased their PPO activity through bioregulation. With the extension of stress duration, the accumulation of phenolics began to decrease, and PPO activity also decreased. PPO activity did not differ significantly among the temperature treatments throughout the stress process (*p* > 0.05).

### Correlations of different physiological indicators

As shown in Table 2, among the 12 physiological indicators, the RC was negatively and highly significantly correlated with SOD, CAT, IAA, and GA. It had a positive correlation with MDA, SS, Pro, and ABA, among which the positive correlation was highly significant with MDA. There were positive correlations among the antioxidant enzyme indicators (SOD, POD, PPO, and CAT), among which CAT was highly significant with SOD and POD. The antioxidant enzymes had a positive correlation with SP, IAA and GA, and SOD had a very significant positive correlation with IAA and GA. The antioxidant enzymes negatively correlated with MDA, SS, Pro, and ABA. Among them, MDA had a very significant negative correlation with SOD, CAT, and a very significant negative correlation between PPO and SS. SP was negatively correlated with SS and extremely negatively correlated with Pro. IAA, ABA, and GA were all highly significantly correlated with each other, with IAA and GA highly significantly positively correlated and ABA highly significantly negatively correlated with the other two.

**Table 2.** Correlation Analysis Among Physiological Indicators in Yunwu Tribute Tea

	SOD	POD	PPO	CAT	MDA	SS	SP	Pro	IAA	ABA	GA
RC	−0.813^**^	*−0.127*	*−0.04*	−0.623^**^	0.679^**^	**0.068**	*−0.206*	**0.344**	−0.594^**^	**0.4**	−0.621^**^
SOD		**0.115**	**0.226**	0.580^**^	−0.634^**^	*−0.157*	0.555^*^	−0.555^*^	0.628^**^	−0.459^*^	0.589^**^
POD			**0.144**	0.469^*^	*−0.223*	*−0.077*	**0.085**	*−0.189*	**0.218**	*−0.383*	**0.292**
PPO				**0.19**	*−0.264*	−0.640^**^	0.490^*^	*−0.213*	**0.06**	*−0.269*	**0.042**
CAT					−0.617^**^	*−0.226*	**0.133**	*−0.009*	**0.34**	*−0.017*	**0.367**
MDA						**0.268**	−0.485^*^	0.499^*^	−0.549^*^	0.480^*^	−0.492^*^
SS							−0.471^*^	**0.265**	*−0.073*	0.529^*^	*−0.144*
SP								−0.677^**^	**0.207**	−0.501^*^	**0.157**
Pro									−0.448^*^	0.678^**^	−0.491^*^
IAA										−0.636^**^	0.915^**^
ABA											−0.645^**^

The bold represents a positive correlation, and the italic represents a negative correlation.

^*^ *p* < 0.05; ^**^p < 0.01.

ABA, abscisic acid; CAT, catalase; GA, gibberellin; IAA, indoleacetic acid; MDA, malondialdehyde; POD, peroxidase; Pro, proline; RC, relative conductivity; SOD, superoxide dismutase; SP, soluble protein; SS, soluble sugars.

### The relationship between gene expression and corresponding physiological indicators

**FIG. 13.**
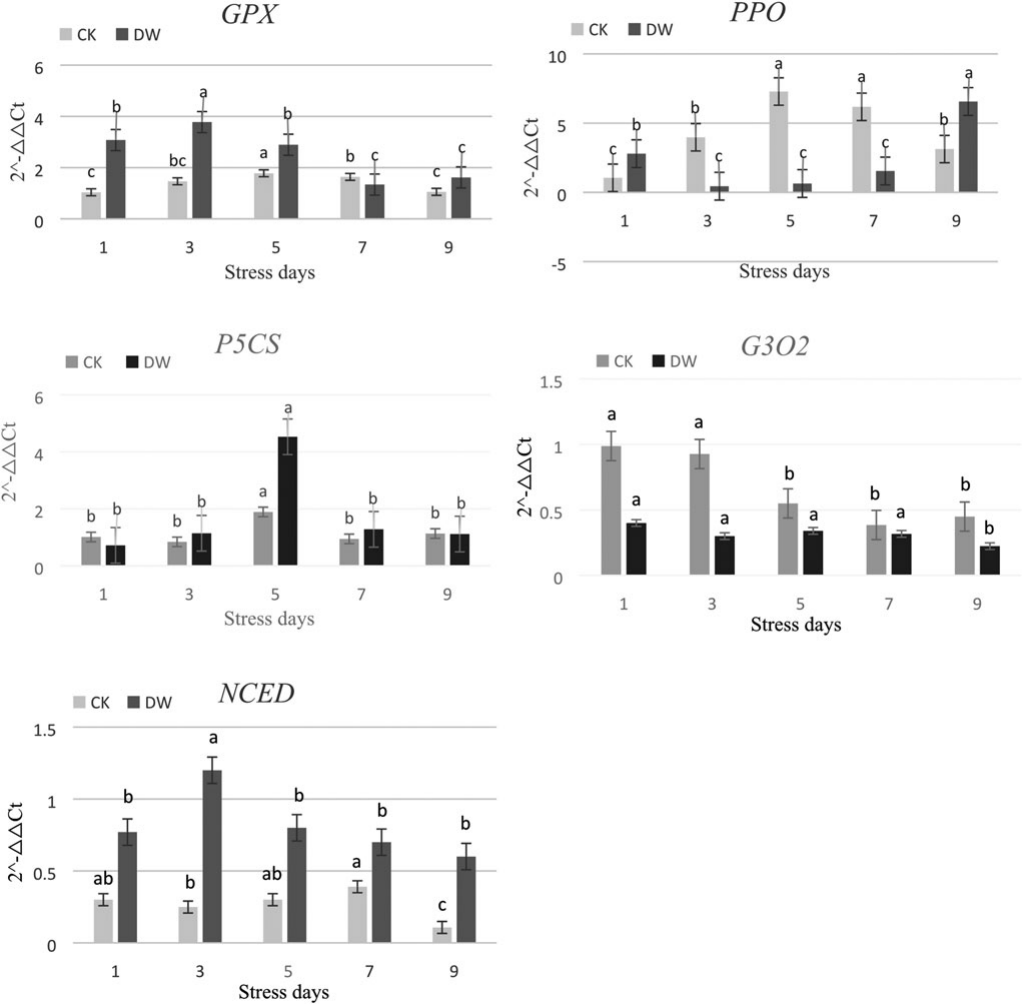
Expression of studied genes in tea plants exposed to durative cold stresses detected by qRT-PCR. CK is the control group and DW is the cold-treated group: (CK: 25°C; DW: 1°C). Data are displayed as mean values of the three replicates with standard error. The different letters indicate significant difference at 0.05 level among different samples. *GPX*, glutathione peroxidase; *P5CS*, Δ1-pyrroline-5-carboxylate synthetase; *G3O2*, gibberellin 3β-dioxygenase; *NCED*, 9-cis-epoxycarotenoid dioxygenase; qRT-PCR, quantitative real-time polymerase chain reaction.

Under cold stress, the levels of expression of *GPX*, *P5CS*, and *NCED* were significantly upregulated in Yunwu Tribute Tea, and the level of gene expression first increased and then decreased with the prolongation of the stress duration. Under cold stress, the levels of expression of *PPO* and gibberellin 3β-dioxygenase (*G3O2*) were significantly downregulated in the plant, while the level of expression of *PPO* decreased followed by an increase, and the levels of expression of *G3O2* did not change significantly. The levels of expression of the five candidate genes (*GPX*, *PPO*, *P5CS*, *G3O2*, and *NCED*) under cold stress were consistent with the changes in the corresponding physiological indicators (Fig. 13).

At low temperatures, the levels of expression of *GPX*, *PPO*, and *G3O2* were significantly and positively correlated with the activities of POD and PPO and the content of GA (*p* < 0.01). The level of expression of *P5CS* significantly and positively correlated with the content of Pro (*p* < 0.05). The level of expression of *NCED* significantly and positively correlated with the content of ABA in the DW but not in the CK (Table 3).

**Table 3.** Correlation Coefficients for the Levels of Gene Expression and the Corresponding Physiological Indicators

	DW					CK				
	POD	PPO	Pro	GA	ABA	POD	PPO	Pro	GA	ABA
*GPX*	0.92^**^					0.91^**^				
*PPO*		0.93^**^					0.94^**^			
*P5CS*			0.73^*^					0.76^*^		
*G3O2*				0.81^**^					0.86^**^	
*NCED*					0.89^**^					−0.45

CK is the control group and DW is the cold-treated group: (CK: 25°C; DW: 1°C).

^*^ *p* < 0.05; ^**^p < 0.01.

## Discussion

### Effects of cold stress on the activity of antioxidant enzymes in the tea plants

As one of the most important antioxidant enzymes in cells, SOD is the first line of intracellular defense against reactive oxygen species (ROS), and its main function is the disproportionation of the deleterious superoxide anion (O^2−^) to produce harmless O_2_ and H_2_O_2_ (Yu *et al.*, 2010). In this experiment, the activity of SOD increased and then decreased under the low-temperature treatment of 4°C and 1°C. The highest value appeared at 3 days after 1°C treatment, indicating that the plant could improve its ability to adapt to cold stress through the increase in enzyme activity within a particular low-temperature range, while the ability of the plant to tolerate cold had been exceeded after 3 days treatment at 1°C. Thus, the ability of plant to effectively resist cold stress had dissipated.

Under different temperatures, the MDA content increased. After 3–9 days of treatment at the low temperature of 1°C, the MDA content was significantly higher than that of the other treatments, and the cell membrane was most severely damaged. This may be owing to the fact that the decrease in the activities of SOD, POD, CAT, and other protective enzymes may cause the accumulation of harmful free radicals and even exceed the damage threshold, which may directly or indirectly initiate membrane lipid peroxidation and increase the molar concentration of MDA. In turn, the accumulation of MDA inhibits the activities of SOD, POD, and CAT, further reducing the ability of antioxidant enzymes to scavenge active oxygen, resulting in increased damage to the membrane system (Ye, 1994).

Studies have shown that PPO has a close relationship with various types of stress resistance of plants. In this study, the activity of PPO showed a trend of increasing and then decreasing during the entire stress process at all temperatures. In the early stage of the stress treatment, the PPO activity was enhanced by the biological regulation of plants to resist the low temperatures.

### Effects of cold stress on tea plant osmotic adjustment substances

The tea plant has antifreezing physiological and biochemical mechanisms. The contents of proline, SP, and soluble sugar of fresh tea leaves all increased and then decreased with the decrease in temperature; however, the temperature range of different varieties of tea plants in response to cold stress differed (Huang *et al.*, 2014). Some studies have shown that the frost resistance of tea plant is closely related to temperature, and the contents of soluble sugar, proline, and SP of fresh tea leaves are closely related to their ability to resist frost (Lin *et al.*, 2015).

In this study, the contents of SP, proline, and soluble sugar of Yunwu Tribute Tea under different temperature treatments were basically higher than those of the control, and only the SP was lower than the control after 3 days treatment at 1°C. Under each temperature treatment, the content of three indicators increased first and then decreased with the prolongation of stress duration.

This indicated that soluble substances were related to plant cold resistance in a particular range. When the temperature or stress duration exceeded the maximum range that the plant could tolerate, the synthesis of soluble substances decreased, and its protective effect also decreased (Zhang *et al.*, 2013).

### Effects of cold stress on endogenous hormones in tea plants

Plant hormones interact with each other in biological signaling processes, including biosynthetic, metabolic, transportation, and signal transduction pathways (Cheng *et al.*, 2013; Du *et al.*, 2013; Golenberg and West, 2013; Miransari and Smith, 2014). In recent years, ABA has been recognized as a stress-growth regulator that plays an important role in the regulation of resistance of plants to cold and the maintenance of structural function of cell membranes. After the plants have been subjected to biotic and abiotic stresses, the endogenous content of ABA increases, and the expression of various resistance genes is induced, thus, enhancing their resistance. The increase in content of ABA has a significant effect on the improvement of plant resistance (Cao *et al.*, 2011; Lee and Luan, 2012; Nakamura *et al.*, 2013; Dangquah *et al.*, 2014; Zeng *et al.*, 2016). During the same duration of stress, the content of ABA of Yunwu Tribute Tea tended to increase as the stress temperature decreased, which may be a protective response of tea plants to low temperature. With the increase in duration of stress, the content of ABA tends to decrease and then increase following each treatment with low temperature. This may be owing to the fact that ABA content of this tea could not be continuously synthesized after accumulating to a certain level, and the cell membrane and protective enzyme system of the plant had been damaged to some extent at the end of the stress period.

Auxin was the first type of plant growth regulator discovered, and IAA is a common type of auxin. It regulates lateral root formation, vascular tissue differentiation, apical dominance, and plant tropism (Kosova *et al.*, 2012). With the extension of duration of stress, most of the content of IAA increased first and then decreased. The IAA content of Yunwu Tribute Tea decreased under the low-temperature treatments of 1°C and 4°C compared with that of the control. Under the low-temperature treatment of 10°C, the content of IAA increased compared with that of the control. This was consistent with the response of content of IAA to low temperatures in wheat (Kosova *et al.*, 2012) and dove-tree (*Davidia involucrata*) (Guo *et al.*, 2013). However, some studies have shown that the content of IAA increased following cold stress (Ou *et al.*, 2007), which may be related to the difference between test materials and processing conditions, and the influence of external environmental conditions.

The first plant growth regulators believed to be related to cold resistance are the gibberellins. Studies have shown that the content of gibberellins in plants with strong cold resistance is generally lower than that in plants with weak cold resistance (Luo, 1989). The results of this study showed that under the same stress temperature, the GA content of Yunwu Tribute Tea decreased to differing degrees as the stress duration increased. The 1°C and 4°C treatments were lower than those of the control, and the 10°C treatment was higher than that of the control. This indicates that Yunwu Tribute Tea can adapt to the-low temperature stress by reducing the content of GA and slowing down plant growth.

### Response of cold resistance-related genes in the tea plant to low temperature

Glutathione peroxidase (*GPX*) is an enzyme that scavenges ROS and is encoded by the gene network that is involved in the regulation of ROS. The main role of *GPX* is to participate in the elimination of matrix H_2_O_2_. Many studies have shown that *GPX* is closely related to various stresses and is a stress-inducing gene (Hu *et al.*, 2011; Zhang *et al.*, 2012). *PPO* is related to the leaf growth and development process, primarily to the phenolic content produced during leaf growth and development (Yao and Jia, 1998). Genetic engineering studies confirmed that the overexpression of Δ^1^-pyrroline-5-carboxylate synthetase (*P5CS*) in transgenic tobacco improved its resistance to penetration and cold stresses (Konstantinova *et al.*, 2002). A study (Wang *et al.*, 2018) indicated that the 9-cis-epoxycarotenoid dioxygenase (*CsNCED*) gene plays an important role in the synthesis, metabolism, and stress response of ABA in tea plants. The overexpression of *NCED1* in plants, such as tobacco (*Nicotiana plumbaginifolia*), can increase the cellular content of ABA (Tung *et al.*, 2008; Zhang *et al.*, 2008; Nitsch *et al.*, 2009). *G3O2* is a key enzyme for the mutual conversion of different gibberellins. It belongs to the 2-ketoglutarate-dependent dioxygenase gene family (2ODD), which is the key enzyme for the synthesis and regulation of gibberellins. They are primarily responsible for catalyzing the mutual conversion between different GAs (Hedden and Phillips, 2000).

By comparing and analyzing the levels of expression of five cold-related genes in Yunwu Tribute Tea under low-temperature stress, this study found that the levels of expression of *GPX*, *P5CS*, and *NCED* were significantly upregulated, while the levels of expression of *PPO* and *G3O2* were significantly downregulated.

In this study, the ABA content of Yunwu Tribute Tea significantly positively correlated with the level of expression of *NCED* under cold stress. The level of expression of *NCED* first increased and then decreased with the prolongation of cold stress and reached a peak value on the fifth day of cold stress. It was 4.8 times that of the control and then was significantly downregulated, further indicating that the plant accumulates a low amount of ABA during the late stage of cold stress. The trend of expression of *G3O2* in the plant was similar to that of the GA content, and the two were highly significantly and positively correlated, which confirmed the reliability of GA test results in this study. In addition, experiments with prolonged stress duration showed that the peak levels of expression of *NCED* and *GPX* were significantly earlier than that of *P5CS* under cold stress, further indicating that tea plants preferentially synthesize ABA and POD under cold stress. In this study, the level of expression of *P5CS* significantly and positively correlated with the content of Pro under cold stress. The level of expression of *P5CS* reached a peak on the fifth day of cold stress, which was 2.4-fold higher than that of the control. However, the content of Pro continued to increase, indicating that the tea plant primarily used the *P5CS* pathway to synthesize Pro. After continuous low temperature, the levels of the content of Pro and expression of *P5CS* decreased, indicating that with the extension of stress duration, the self-osmoregulatory effect gradually decreased. Combined with the results of transcriptome sequencing analysis, it further showed that tea plant primarily uses the *P5CS* pathway to synthesize Pro.

Under cold stress, the patterns of expression of the *GPX* and *PPO* genes were consistent with the trends of POD and PPO activities, and both were highly significantly positively correlated, indicating that these two genes play an important role in regulating the activities of POD and PPO.

The changes of physiological indicators of Yunwu Tribute Tea under different cold stress conditions, their correlation, and the levels of expression of their corresponding genes were investigated. These results will help to understand the mechanism of response of Yunwu Tribute Tea to cold stress and thus provide a theoretical basis for mining the cold resistance genes of tea plants.

## Supplementary Material

Supplemental data

Supplemental data

Supplemental data

Supplemental data

Supplemental data

Supplemental data

## References

[B1] Cao, F.Y., Yoshioka, K., and Desveaux, D. (2011). The roles of ABA in plant–pathogen interactions. J Plant Res 124, 489–499 21380629 10.1007/s10265-011-0409-y

[B2] Cheng, Y.Q., Liu, J.F., Yang, X.D., Ma, R., Liu, Q., and Liu, C. (2013). Construction of ethylene regulatory network based on the phytohormones related gene transcriptome profiling and prediction of transcription factor activities in soybean. Acta Physiologiae Plantarum 35, 1303–1317

[B3] Chinnusamy, V., Schumaker, K., and Zhu, J.K. (2004). Molecular genetic perspectives on cross-talk and specificity in abiotic stress signalling in plants. J Exp Botany 55, 225–236 14673035 10.1093/jxb/erh005

[B4] Dangquah, A., Zelicourt, A.D., Colcombet, J., and Hirt, H. (2014). The role of ABA and MAPK signaling pathways in plant abiotic stress responses. Plant Biotechnol 32, 40–52 10.1016/j.biotechadv.2013.09.006 24091291

[B5] Du, H., Wu, N., Chang, Y., Li, X., Xiao, J., and Xiong, L. (2013). Carotenoid deficiency impairs ABA and IAA biosynthesis and differentially affects drought and cold tolerance in rice. Plant Mol Biol 83, 475–488 23846670 10.1007/s11103-013-0103-7

[B6] Golenberg, E.W., and West, N.W. (2013). Hormonal interactions and gene regulation can link monoecy and environmental plasticity to the evolution of dioecy in plants. Am J Botany 100, 1022–1037 23538873 10.3732/ajb.1200544

[B7] Guo, B., Stiles, A.R., and Liu, C.Z. (2013). Changes in endogenous hormones and oxidative burst as the biochemical basis for enhanced shoot organogenesis in cold-treated Saussurea involucrata explants. Acta Physiol Plantamm 35, 283–287

[B8] Gusta LV, Trischuk, R., and Weiser, C.J. (2005). Plant cold acclimation: the role of abscisic acid. J Plant Growth Regul 24, 308–318

[B9] Hao, X., Wang, L., Zeng, J., Yang, Y., and Wang, X. (2018). Response and Adaptation Mechanisms of Tea Plant to Low-Temperature Stress. Stress Physiology of Tea in the Face of Climate Change. (Springer Nature, Singapore) pp. 39–61

[B10] He, Y.J., Xie, L., Ren, X.R., Cao, H., Liang, L.-L., and Xu, Y. (2008). Effects of low temperature stress on physiological characteristics of six tree species seedlings. Chin J Ecol 27, 524–531

[B11] Hedden, P., and Phillips, A.L. (2000). Gibberellin metabolism: new insights revealed by the genes. Trends Plant Sci 5, 523–530 11120474 10.1016/s1360-1385(00)01790-8

[B12] Hildebrandt, T.M., Nunes, N.A., Araújo, W.L., and Braun, H. (2015). Amino acid catabolism in plants. Mol Plant 8, 1563–1579 26384576 10.1016/j.molp.2015.09.005

[B13] Hu, M.L., Pu, H.M., Long, W.H., Gao, J.Q., Chen, X.J., Zhang, J.F., *et al.* (2011). Cloning and expression of glutathione peroxidase 2 gene in rapeseed (*Brassica napus* L.). Jiangsu J Agricult Sci 27, 950–956

[B14] Huang, H.T., Yu, J.Z., Wang, X.B., Zhang, W., Zhou, T.F., and Ao, C. (2014). Study on physiological characters of new shoot in different cold-resistant tea varieties in autumn. Acta Agricult Zhejiangensis 4, 925–928

[B15] Kong, X.S., and Yi, X.F. (2008). Plant Physiology and Physiology. (China Agriculture Press, Beijing) pp. 233–257

[B16] Konstantinova, T., Parvanova, D., Atanassov, A., and Djilianov, D. (2002). Freezing tolerant tobacco, trans-formed to accumulate osmo protectants. Plant Sci 163, 157–164

[B17] Kosova, K., Prasil, I.T., Vitdmv, P., Dobrev, P., Motyka, V., Flokovác, K., *et al.* (2012). Complex phytohormone responses during the cold acclimation of two wheat cultivars differing in cold tolerance, winter Samanta and spring Sandra. J Plant Physiol 169, 567–576 22304971 10.1016/j.jplph.2011.12.013

[B18] Lee, S.C., and Luan, S. (2012). ABA signal transduction at the crossroad of biotic and abiotic stress responses. Plant Cell Environ 35, 53–60 21923759 10.1111/j.1365-3040.2011.02426.x

[B19] Li, H.S. (2000). Plant Physiology and Biochemistry. (Higher Education Press, Beijing) pp. 14–185

[B20] Li, J., Yang, Y., Sun, K., Chen, Y., Chen, X., and Li, X. (2019). Exogenous melatonin enhances cold, salt and drought stress tolerance by improving antioxidant defense in tea plant (*Camellia sinensis* (L.) O. Kuntze). Molecules 24, 1826 10.3390/molecules24091826 PMC6539935 31083611

[B21] Lin, Z.H., Zhong, Q.S., and Chen, C.S. (2015). Advances in resistance breeding of tea tree. Tea Fujian 4, 2–4

[B22] Liu, Y., Qi, X., Wang, Y., Lin, C.H., Wang, J.-H., Sun, C., *et al.* (2012). Classification characteristics of seedling population of Guiding Yunwu Tribute Tea. Guizhou Agricult Sci 39, 33–37

[B23] Luo, Z.R. (1989). Relationship between plant hormones and cold resistance. Mol Plant 3, 1–5

[B24] Miransari II, M., and Smith, D.L. (2014). Plant hormones and seed germination. Environ Exp Botany 99, 110–121

[B25] Nakamura, T., Yazaki, J., Kishimoton, N., Kikuchi, S., Robertson, A.J., Gusta, L.V., *et al.* (2013). Comparison of long-term up-regulated genes during induction of freezing tolerance by cold and ABA in bromegrass cell cultures revealed by microarray analyses. Plant Growth Regul 71, 113–136

[B26] Nitsch, L.M.C., Oplaat, C., Feron, R., Ma, Q., Arts, M.W., Hedden, P., *et al.* (2009). Abscisic acid levels in tomato ovaries are regulated by *LeNCEDl* and SICYP707Al. Planta 229, 1335–1346 19322584 10.1007/s00425-009-0913-7

[B27] Ou, Y.L., Hong, Y.H., Huang, L.H., Wang, R.Z., and Xiao, L.T. (2007). On changes of physiology and biochemistry and plant hormones in super rice seedlings by different stress signaling. Res Agricult Modern 28, 104–106

[B28] Ozkur, O., Ozdemir, F., Bor, M., and Ismail, T. (2009). Physiochemical and antioxidant responses of the perennial xerophyte Capparis ovata Desf. to drought. Environ Exp Bot 66, 487–492

[B29] Shen, Y.M, Ma, J., Huang, Y.Y, and Jiang, X. (2009). Effect of cold stress on membrane permeability of five evergreen shrubs for Landscape. J Zhejiang Forestry Sci Technol 28, 28–31

[B30] Tung, S.A., Smeeton, R., White, C.A., Black, C., Taylor, I.B., Hilton, H.W., and Thompson, A.J. (2008). Over-expression of *LeNCEDl* in tomato (*Solanum lycopersicum* L.) with the rbcS3C promoter allows recovery of lines that accumulate very high levels of abscisic acid and exhibit severe phenotypes. Plant Cell Environ 31, 968–981 18373621 10.1111/j.1365-3040.2008.01812.x

[B31] Wang, Y., He, H.Z., Liu, Y., Wang, J.H., and Sun, C. (2013). A preliminary study on the tissue culture of Yunwu Tribute Tea. Guizhou Sci 31, 95–96

[B32] Wang, Y., Luo, G.K., and Sun, C. (2011). Study on annual variation of enzyme and amino acid content of Yunwu tea in guiding by light. Jilin Agricult 255, 138–139

[B33] Wang, Z., Chen, D., Yue, C., Cao, H.L., and Guo, Y.L. (2018). Cloning and expression analysis of CsNCED2 Gene in tea plant (*Camellia sinensis*). Acta Bot Boreali Occidentalia Sin 38, 0994–1002

[B34] Weyers, J.D.B., and Paterson, N.W. (2002). Plant hormone and the control of physiological processes. New Phytol 152, 375–407 10.1046/j.0028-646X.2001.00281.x 33862994

[B35] Wu, S.D. (2008). Rare low temperature rain and snow weather will cause production of spring tea spring tea market delay. Chinese Tea World 2, 8

[B36] Yao, Y., and Jia, K.X. (1998). Study on the Relationship of Copper and Polyphenol oxidase in Larix principis-rupprechtii. Chin Sci Abstr 19, 305–308

[B37] Ye, F.X. (1994). Comparison of polyphenol oxidase activity between different ages of Lentinus edodes. Edible Fungi Jiangsu 15, 29–30

[B38] Yu, W.W, Cao, F.L., and Wang, G.B. (2010). Relationship between active oxygen metabolism of ginkgo leaf and cell membrane injury under low-temperature stress. J Northeast Forestry Univer 38, 46–48

[B39] Zeng, G.H., Ma, Q.P., Wang, W.D., Zhou, L., Yin, Y., and Li, X.H. (2016). Effect of natural low-temperature on endogenous hormones of *Camellia sinensis* (L.) Kuntze Plant. J Tea Sci 36, 85–91

[B40] Zhang, L.L., Xu, B.Y., Liu, J.H., Jia, C.H., Zhang, J.B., Wang, J.S., *et al.* (2012). Isolation and expression analysis of a cDNA encoding glutathione peroxidase from banana. Acta Horticult Sin 39, 1471–1481

[B41] Zhang, Y., Yang, J.E., Lu, S.Y., Cai, J.L., and Guo, Z.F. (2008). Overexpressing *SgNCED1*in tobacco increases ABA level, antioxidant enzyme activities, and stress tolerance. J Plant Growth Regul 12, 151–158

[B42] Zhang, Y.H., Rong, J.D., Li, S.P., He, T.Y., Chen, L.Y., and Zheng, Y.S. (2013). Effect of low temperature on biochemical characteristics of Prunus campanulata Maxim. J Fujian Coll Forestry 33, 326–329

[B43] Zhu, Q.W, Fan, K., Xie, Y.L., Dong, J.F., Zhan, Y.W., and Luo, Y.P. (2013). Progress in plant cold-stress-responsive miRNAs and the application in cold resistance research of *Camellia sinensis*. J Tea Sci 33, 212–220

[B44] Zou, Z.W, Fang, W.P., Zhang, D., Duan, Y.S., and Li, X.H. (2008). Analysis of differential expression genes in cold-induced tea plant. J Tea Sci 28, 249–254

