# Supplementary material for: Physiological Changes and Differential Gene Expression of Tea Plants (Camellia sinensis (L.) Kuntze var. niaowangensis Q.H. Chen) Under Cold Stress
Source: DNA Cell Biol. 2021 Jul 15;40(7):906–20. doi: 10.1089/dna.2021.0147 (PMC8309439; doi:10.1089/dna.2021.0147)
Supplement: Supplemental data [file Supp_Fig5.doc]

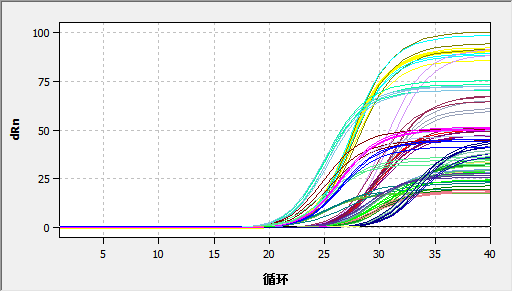

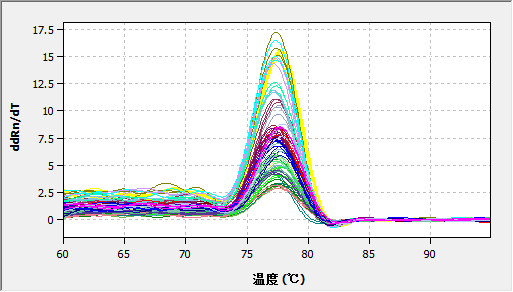


Fig.5 The image of target gene *NCED*. The left is the amplification curve,abscissa is the number of cycles.The right is the melting peaks, abscissa is the dissolution temperature,the product TM value is 77℃.
